# Global Distribution of Two Fungal Pathogens Threatening Endangered Sea Turtles

**DOI:** 10.1371/journal.pone.0085853

**Published:** 2014-01-21

**Authors:** Jullie M. Sarmiento-Ramírez, Elena Abella-Pérez, Andrea D. Phillott, Jolene Sim, Pieter van West, María P. Martín, Adolfo Marco, Javier Diéguez-Uribeondo

**Affiliations:** 1 Departamento de Micología, Real Jardín Botánico-CSIC, Madrid, Spain; 2 Departamento de Biología de la Conservación, Estación Biológica de Doñana-CSIC, Sevilla, Spain; 3 Faculty of Science, Asian University for Women, Chittagong, Bangladesh; 4 One Health Research Group, School of Public Health, Tropical Medicine and Rehabilitation Sciences, James Cook University, Townsville, Queensland, Australia; 5 Conservation Office, Ascension Island Government, George Town, Ascension Island; 6 Aberdeen Oomycete Laboratory, College of Life Sciences and Medicine, University of Aberdeen, Scotland, United Kingdom; Uppsala University, Sweden

## Abstract

Nascent fungal infections are currently considered as one of the main threats for biodiversity and ecosystem health, and have driven several animal species into critical risk of extinction. Sea turtles are one of the most endangered groups of animals and only seven species have survived to date. Here, we described two pathogenic species, *i.e., Fusarium falciforme* and *Fusarium keratoplasticum*, that are globally distributed in major turtle nesting areas for six sea turtle species and that are implicated in low hatch success. These two fungi possess key biological features that are similar to emerging pathogens leading to host extinction, *e.g.*, high virulence, and a broad host range style of life. Their optimal growth temperature overlap with the optimal incubation temperature for eggs, and they are able to kill up to 90% of the embryos. Environmental forcing, *e.g.*, tidal inundation and clay/silt content of nests, were correlated to disease development. Thus, these *Fusarium* species constitute a major threat to sea turtle nests, especially to those experiencing environmental stressors. These findings have serious implications for the survival of endangered sea turtle populations and the success of conservation programs worldwide.

## Introduction

In the last decades, fungal pathogens have been found to cause the major losses and even extinctions of several animal species [1]. There is an increasing number of unknown fungi or fungal-like species being reported as responsible for high-profiled declines in animal wildlife. Examples are *Geomyces destructans* in bats [2], the amphibian skin infecting fungus *Batrachochytrium dendrobatidis* [3], *Aspergillus sidowii* in soft corals [4] the microsporidian fungus *Nosema* sp in bees [5], and the oomycete *Aphanomyces* spp. in freshwater crayfish and fish [1]. In this study, we identified two novel fungal pathogens responsible for a previously unknown disease of one of the most threatened groups of vertebrates, *i.e.*, sea turtles. We present data showing that these pathogens are distributed over a broad geographic area, have a broad host range, and demonstrate high virulence on sea turtle eggs. These biological features can contribute to contemporary increases in disease emergence and host extinction.

Sea turtles are relic animals from the Triassic period of more than 210 million years ago. Today, they are among the largest reptiles in the world and one of the most endangered animals [6]. Most of the seven sea turtle species show a population decline of as high as 30–80% [7]. The main known causes of declining populations include the intensive activities of the fishing industry, habitat deterioration, pollution by plastic debris, destruction of nesting areas by human activities, hunting of adults and consumption of eggs, predators, and pathogens [7]–[9]. Pathogens, such as the virus causing fibropapillomatosis, have been implicated in serious decline of some green sea turtle populations [10].

The fungal pathogen *Fusarium solani* (Mart.) Saccardo (1881) has also been found in sea turtle eggs [8], [11]–[19], and recent reports have shown that this pathogen is associated with mass mortalities in natural and relocated nests of the sea turtle species, *Caretta caretta* in Boa Vista, Cape Verde [9]. The detected fungus is actually a monophyletic “species complex” *i.e.*, *Fusarium solani* species complex, FSSC, which includes over 60 phylogenetic species [20]–[22]. This complex is widely distributed, and comprises soil-borne saprotrophs that are among the most frequently isolated fungal species from soil and plant debris. The phylogenetic relationships and geographical distribution of *F. solani* species responsible for important human and plant diseases have been studied [23], [24]. However, *F. solani* isolates from failed eggs of all sea turtle species have not been similarly investigated.

## Results and Discussion

### Worldwide *Fusarium*-disease survey and pathogen isolations

**Figure 1:**
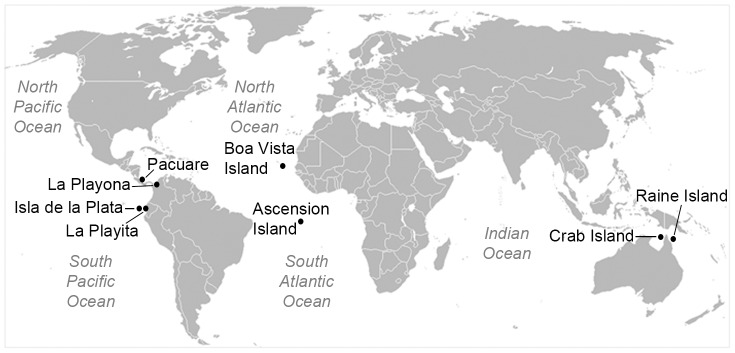
Sea turtle-nesting regions sampled for the presence of *Fusarium*. Eggshells from a total of six species of sea turtle were collected from some of the main nesting beaches in the Atlantic, Indian and Pacific Oceans, and the Caribbean Sea, *i.e.*, *Chelonia mydas* (Raine Island, Australia; Isla de la Plata at Machalilla National Park, Ecuador; and Ascension Island), *Caretta caretta* (Boa Vista Island, Cape Verde), *Eretmochelys imbricata* and *Lepidochelys olivacea* (La Playita, Machalilla National Park), *Dermochelys coriacea* (La Playona, Colombia and Pacuare Nature Reserve, Costa Rica), and *Natator depresus* (Crab Island, Australia).

**Figure 2:**
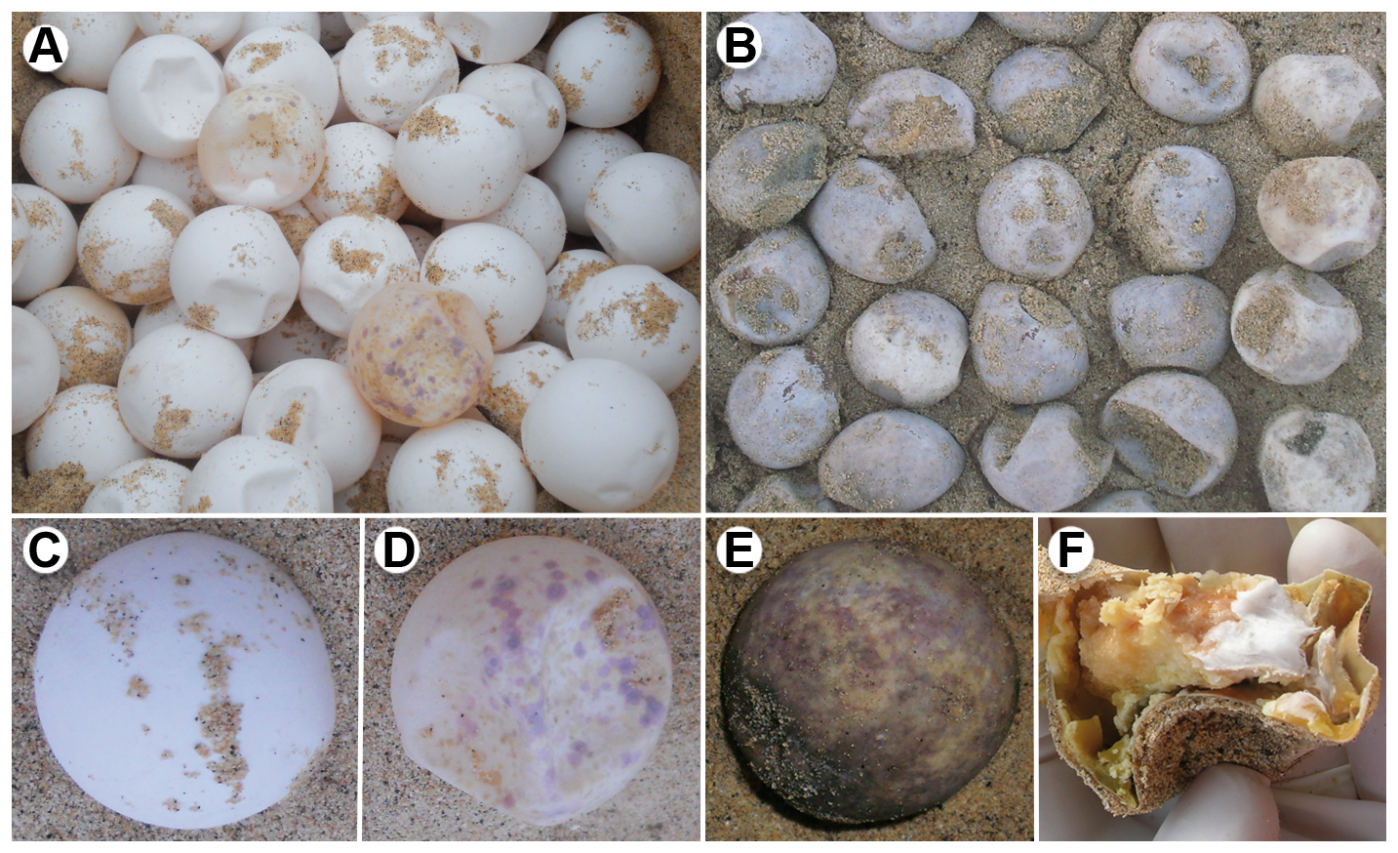
Nests of the sea turtle species *Caretta caretta* affected by *Fusarium* at different stages of incubation. (A) Nest at initial stage of incubation (two weeks) showing an egg with characteristic signs of *Fusarium* infection (yellowish, reddish and bluish spots). (B) Nest at final stage of incubation (eight weeks) with all eggs with advanced signs of *Fusarium* infection. (C–D) Eggs at early stage of incubation. (C) Healthy asymptomatic egg. (D) Symptomatic egg. (E–F) Eggs at late stage of incubation. E) Egg with severe signs of *Fusarium* disease. F) Dead embryo on *Fusarium* infection with white mycelia inside the egg.

In order to determine the incidence of *F. solani* in sea turtle nests, we conducted a disease survey of six of the major sea turtle-nesting regions in the Atlantic, Indian, and Pacific Oceans, and the Caribbean Sea during the period from 2005 to 2012 (Figure 1). *Fusarium*-colonized eggs were found in all sea turtle species and nesting areas surveyed. We sampled eggs with and without macroscopic signs of *Fusarium* infection from different stages of embryonic development (Figure 2) and obtained 119 fungal isolates that were initially identified as *F. solani* based on their morphological characters [25], [26] and the BLAST search of their ITS nrDNA sequences. This fungal species was never isolated from eggs taken directly from the ovipositor of nesting females (prior to contact with the sand), nor from the cloacal mucus, suggesting a sand-born origin. Moreover, a progressive colonization of the eggs in the turtle nests was indicated by the increase in numbers of eggs with signs of *Fusarium* infection and signs from early to advanced stages of incubation (Figure 2A, B) and the isolation of *F. solani* from all the stages tested (Table S1).

The phylogenetic tree based on Maximum Parsimony and Bayesian phylogenetic analyses (Figures S1) of 119 *F. solani* sea turtle isolates (STI) and 62 selected sequences of *Fusarium* spp. from other hosts, resulted in all STI grouping in two subclades separated from the majority of isolates of the FSSC so far described [20], [21], [23], [27]. Thus, the sequences from selected *Fusarium* spp. grouped in three separate clades of the FSSC that were named clade I, II and III. The clade I contained isolates of previously described species *F. illudens* and *F. plagianthi*. The clade II grouped isolates, previously characterized as *F. solani* formae speciales, that have been recently renamed and formally described as new species [28], [29]. The clade III comprised three subclades (A, B and C). The subclade B predominantly grouped sequences of plant pathogenic isolates of *F. solani* that were previously described by O'Donnell [20] and included the strain for which a full genome sequence has become available [27] (Figure S1). However, the novel STI only clustered in two distinct subclades, that were named A and C. Subclade A contained 46 out of 119 STI, mainly isolated from eggs with signs of *Fusarium* infection, and grouped with reference sequences for *F. falciforme*. Isolates of this group were found in all sea turtle species and nesting areas investigated. In recent years, this species has been described as an emerging animal pathogen [30] but has not been previously reported from sea turtle eggs. Subclade C comprised the majority of the STI, *i.e.*, 79 out of 119, but also GenBank sequences from three *F. keratoplasticum* isolates from humans. Isolates of this group were found in all sea turtle species, except for *D. coriacea* and *L. olivacea*, and all nesting areas sampled, except for the Australian beaches (Figure S1).

### Discovery of two pathogenic species to sea turtle eggs

**Figure 3:**
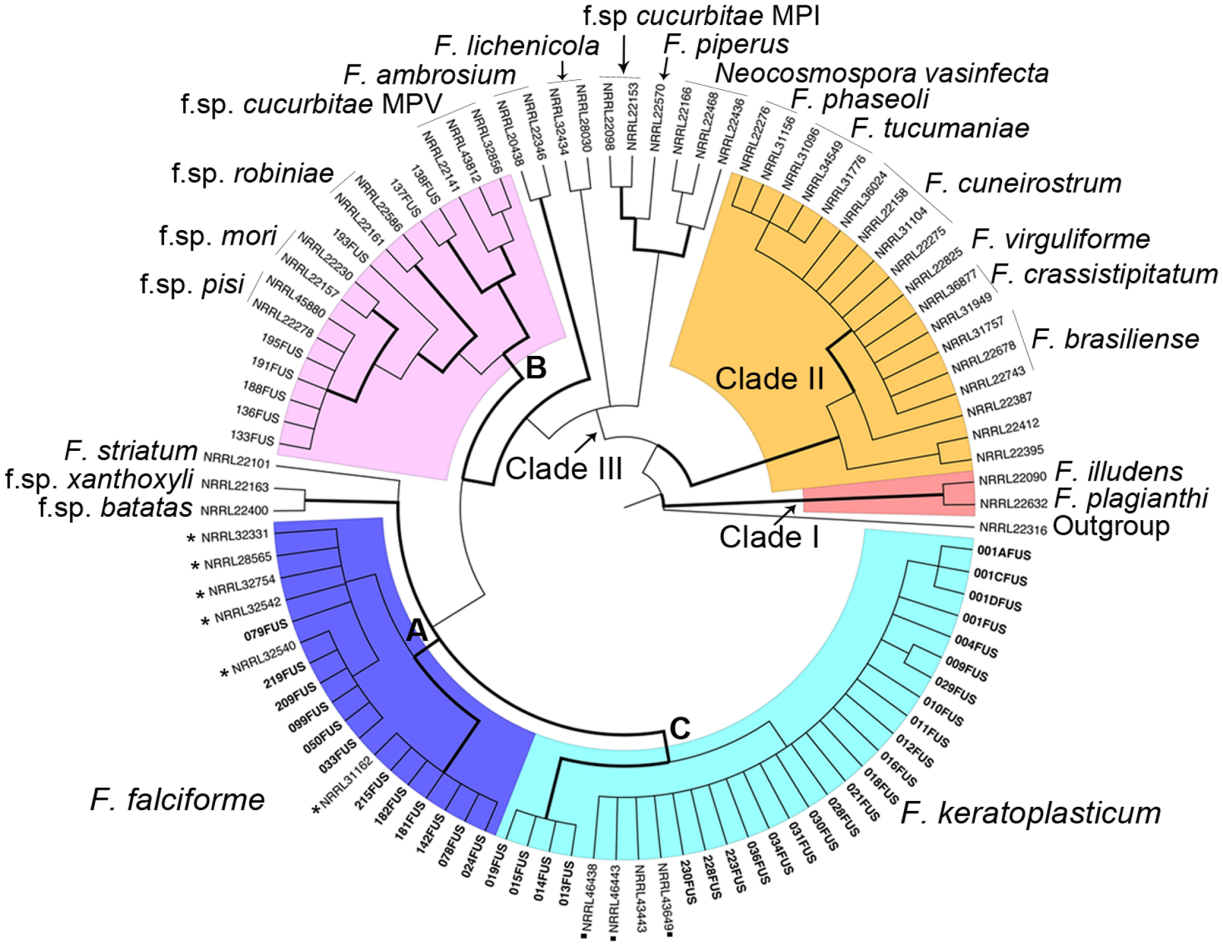
Bootstrapped multilocus out-group-rooted cladogram. Summarized cladogram of the *Fusarium solani* species complex inferred from the DNA regions: ITS nrDNA, LSU nrDNA and *RPB2*. Thick branches correspond to highly supported groups, *i.e.*, posterior probability (PP) and the bootstrap values (BS) of the parsimony and Bayesian analysis when PP≥0.95 and BS≥70. The sea turtle isolates are highlighted in bold. A solid asterisk to the right of an NRRL number identifies the *F. falciforme* isolates. A solid rectangle to the right of an NRRL number identifies *F. keratoplasticum* isolates. For full details of three, see Figure S2.

Since FSSC comprises several phylogenetic species, we conducted combined analyses of three loci to identify the phylogenetic species of these pathogenic *F. solani* STI. This type of analyses has recently allowed a better characterization of the phylogenetic species of the FSSC [23]. Our phylogenetic analyses were based on two loci of the nrDNA, *i.e.*, the internal transcribed spacer (ITS) and part of the nuclear large subunit (LSU), and the second largest subunit of the RNA polymerase II (*RPB2*) gene, of 37 selected STI from worldwide and six turtle species, and of 62 *Fusarium* spp. from other hosts (Tables S1, S2). The combined phylogenetic tree based on Maximum Parsimony and Bayesian phylogenetic analyses (Figures 3, S2) was congruent with that derived from the ITS nrDNA sequences. Thus, the combined analyses resulted in all STI grouping in two subclades separated from the majority of isolates of the FSSC so far described [20], [21], [23], [27]. The sequences from selected *Fusarium* spp. grouped in three highly supported clades of the FSSC that were named clade I, II and III. In these clades, the sequences of the selected *Fusarium* spp. and formae speciales clustered as described above for ITS nrDNA region. The novel STI only clustered in two highly supported distinct subclades of clade III, that were named A and C. Subclade A contained 12 out of 37 STI, mainly isolated from eggs with signs of *Fusarium* infection, and grouped with reference sequences for *F. falciforme*. Isolates of this group were found in Cape Verde, Ecuador and Australia, and in all sea turtle species investigated except for *D. coriacea*. Subclade C comprised the majority of the STI, *i.e.*, 25 out of 37, but also GenBank sequences from three *F. keratoplasticum* isolates from humans. Isolates of this group were found in all sea turtle species investigated, except from *D. coriacea* and *L. olivacea*, and all nesting areas sampled, except for Australia and Costa Rica. The morphological characters of the STI clustering with the species *F. falciforme* and *F. keratoplasticum* were generally consistent with those described for the species [22], [30] (Figure S3). Thus, *F. keratoplasticum* isolates showed ellipsoidal (aseptate) to fusoid microconidia (1–3 septate), mainly produced in long and occasionally branched monophialides. The scarce chlamydospores were smooth-walled, mainly terminal in hyphae, and mostly single of globose type (Figure S3A–C). The *F. falciforme* isolates had ellipsoidal to reniform microconidia (0–3 septate), produced mainly in long, septate, sometimes branched monophialides. Although infrequently observed, *F. falciforme* produced chlamydospores, mainly intercalary in hyphae, single or in chains, globose type, and smooth walled (Figure S3).

### Biological features of the new pathogenic species to sea turtle eggs

These new fungal pathogenic species possess a number of biological features that are identical/similar to those of known fungal pathogens involved in emerging infectious diseases and host extinctions, *i.e.*, high virulence and generalist and opportunistic nature [1]. Thus, both *F. falciforme* and *F. keratoplasticum* where the only species that were consistently isolated from dead embryos (Figure 2E, F; Table S1). Furthermore, in a previous study, STI that belong to the species *F. keratoplasticum* were shown to follow Koch's postulates [9] and have high virulence that can result in mortality rates approaching 100% under controlled conditions [9]. In experiments conducted under hatchery conditions, we found a significant (P<0.01) linear regression relationship (R^2^ = 0.87) of the number of symptomatic *Fusarium*-colonized eggs on the embryonic mortality rates in nests, which reached up to 92% (Figure S4).

**Figure 4:**
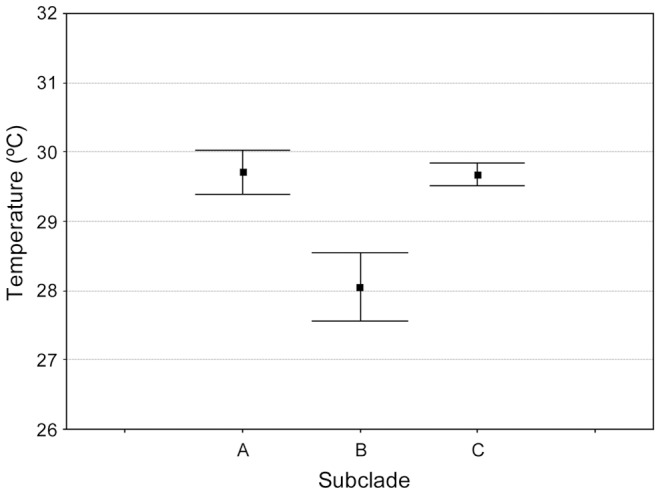
Mean optimal growth temperature (OGT) of *Fusarium* isolates. The OGT was calculated for selected isolates from the sea turtle pathogenic species clustered in subclades A (*Fusarium falciforme*) and C (*Fusarium keratoplasticum*) and non- sea turtle isolates clustered in subclade B, within the *Fusarium solani* species complex. Differences in OGT for each group of isolates were determined by one-way ANOVA followed by a *post-hoc* Tukey HSD test. Data represent mean ± standard error bars (s.e.m) of three independent experiments.

In their environmental niches, fungi have to cope with fluctuations in temperature, pH, osmolarity and other physical stressors [31]. These pathogens show also an important adaptation to the host environment. The range of optimal growth temperature (OGT) was 29.7°C (±1.2) for the species *F. falciforme*, 29.7°C (±0.7) for the species *F. keratoplasticum* and 28°C (±1.5) for isolates belonging to subclade B of the FSSC (Figure 4). No significant differences were found on the media OGT among isolates from *F. falciforme* and *F. keratoplasticum* (Anova, P>0.05). The OGT of these species differed significantly to the OGT of the isolates from subclade B (Anova, P<0.05). Thus, the range of optimal growth temperature (OGT) for the pathogenic species coincided with the range of temperatures suitable for the development of sea turtle embryos (Table 1) [32]–[34]. Moreover, the mean OGT for both fungal species overlapped with the range of pivotal temperature for incubation of sea turtle eggs, *i.e.*, characteristic of temperature-dependent sex determination that reflects the mean temperature of incubation in life history [35] (Figure 4). This property makes optimal conditions for egg incubation to be ideal for pathogen growth and possibly colonization. Similar adaptations to the host environment have also been described in other fungal or fungal like-pathogen systems [36]–[38].

**Table 1:** Mycelial growth at different temperatures of isolates belonging to the *Fusarium solani* species complex (FSSC).

		Micelial growth (mm/day)	
Temperature (°C)	*F. Falciforme* (Subclade A)	Subclade B	*F. keratoplasticum* (Subclade C)
21	8.19 (+0.82)	7.53 (+0.79)	7.80 (+0.65)
25	10.15 (+0.46)	9.23 (+1.61)	9.29 (+0.71)
27	10.90 (+0.70)	10.40 (+1.21)	9.98 (+0.88)
29	12.48 (+1.24)	10.30 (+1.42)	11.20 (+1.11)
32	11.77 (+1.40)	8.20 (+2.79)	10.22 (+1.31)
37	5.66 (+1.02)	2.71 (+1.02)	4.71 (+0.68)

Selected sea turtle isolates (STI) and non-sea turtle isolates were tested. The STI belonged to the two species, *i.e.*, *F. falciforme* (subclade A, *n* = 12) and *F. keratoplasticum* (subclade C, *n* = 12), and the NSTI isolates belonged to subclade B (*n* = 12). The data represent the mean ± SD of three independent experiments.

In addition, both pathogenic *Fusarium* species possess the ability to survive independently outside their host by living as saprotrophs. The sand-born origin of infection was confirmed since only eggs in contact with sand could become infected. Furthermore, these new pathogens show a generalist and opportunistic mode of life as the majority of fungal Ascomycetes. Thus, they could infect not only eggs of all sea turtle species tested but also potato tubers, since typical symptoms of *Fusarium* dry rot disease were observed in inoculated tubers, *i.e.*, dark depressions in the surface of the tubers and necrotic dry tissue along the wounds (Figure S5). The phylogenetic analyses of GenBank sequences also showed the existence of unknown *Fusarium* sequences that cluster with either *F. falciforme* or *F. keratoplasticum* and that were reported in disease crops, animals, and humans (Tables S1, S2). More importantly, conditions leading to disease development seem to be related to environmental factors that allow this opportunistic pathogens to become serious disease as discuss next.

### Environmental change as a driver of fungal infection

**Figure 5:**
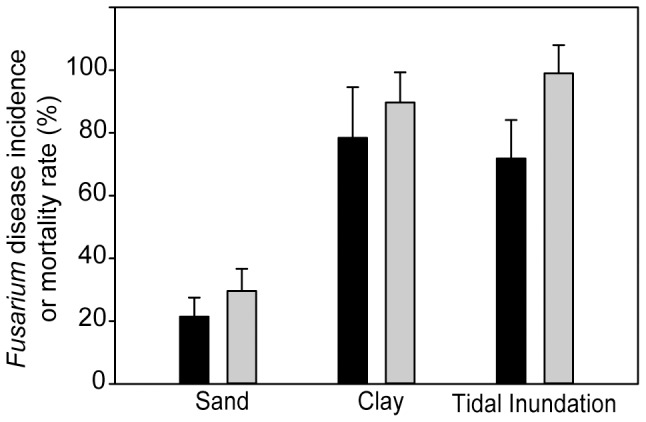
Environmental factors involved on embryonic mortality rate and *Fusarium*-disease incidence. The embryonic mortality rate and *Fusarium* disease incidence were obtained from nests of the sea turtle species *Caretta caretta* in Boa Vista, Cape Verde. Statistics were determined by one-way ANOVA (p>0.01) followed by a *post-hoc* Tukey HSD analysis. Columns represent the average of the *Fusarium* disease incidence (black column) and mortality rate (grey columns) ± standard error bars (s.e.m).

Although the dynamics of the *Fusarium*-disease in sea turtle eggs have been shown to follow Koch's postulates [9], the influence of environmental forcing on disease progression has not been studied before. Interestingly, we observed that nests located in regions prone to tidal inundations, or with contents of clay/silt, exhibited higher embryonic mortality rates and signs of invasion by *Fusarium* than nests in sandy nesting regions not exposed to such stressors. Mortality rates at nesting regions with contents of clay/silt or exposed to tidal inundation were significantly (P<0.05) higher (89.7% and 99.7%, respectively) than those observed in nests not experiencing either variable (29.6%) (Figure 5). The hatch success of both inundated nests and those with clay/silt were severely reduced, and no significant differences in mortality rates were found between them. Moreover, while monitoring nests of *C. caretta* in Cape Verde, the incidence of *Fusarium* disease in nests exposed to inundation or containing clay/silt was significantly higher (78.4% and 71.8% respectively) than that in dry sand nests (21.4%) (Figure 5). Both inundated and clay/silt nests seem to be equally affected since no significant differences in *Fusarium* incidence was found between them.

These observations suggest that environmental forcing seem to represent a key factor for *Fusarium* disease development and hatch-failure in sea turtle eggs. Thus, under conducive conditions for disease development, hatch-failure in nests can be very high due to the infection by these fungal pathogens. Indeed, these factors would favor fungal colonization and disease development, probably by impeding gas exchange through the eggshell to the embryo leading to embryonic stress or death. In addition, dead eggs may serve as a focus of colonization by pathogenic *Fusarium* spp. in the nest, leading to massive production of spores and building inoculum units that allow rapid colonization of live eggs [39]. Similar disease dynamics have been reported for the oomycete *Saprolegnia diclina*, which is a fungal-like pathogen of fish and amphibian eggs [40], [41]. The effects of this *Fusarium* disease can, however, be easily overlooked and underestimated since noticing hatch-failure requires marking and exhumation of the nests.

In summary, we show that the presence of *F. falciforme* and *F. keratoplasticum*, constitute an additional threat to sea turtle nests specially at vulnerable beaches, *e.g.*, with frequent tidal inundation or unsuitable substrates. In future scenarios with nesting beaches affected by climate change, sea turtle nests are very likely to be exposed to further environmental stressors favoring colonization by these pathogenic *Fusarium* spp., including increased tidal wash and sea spray [42], [43], beach erosion and changes in sand composition. Understanding the environmental conditions that favor colonization of sea turtle eggs by these pathogenic *Fusarium* species will allow us to identify nesting regions that may be at risk of infection, and also determine the conditions favorable for *in situ* and *ex situ* incubation (beaches and hatcheries respectively). Current ongoing programs based on *in situ* and *ex situ* incubation are being implemented in important nesting regions throughout the world [44]. These programs increase the numbers of hatchlings produced each year, and they need to be aware of the risk of *Fusarium* disease, so that efforts can be focused on rescuing eggs from risk areas for fungal infection.

## Materials and Methods

### Ethics statement

Collection of sea turtle eggshells and dead embryos were done under permissions: ES-DE-00010/08C and ES-DE-00003/091 for Cape Verde; 002-RM-DPM-M and 0003-EXP-CIEN-RM-DPM-MA for Ecuador; 009-2008-INV-ACLAC for Costa Rica; Research Permit 2801 of the Instituto de Investigaciones Ambientales del Pacífico-Colombia, and EBD-CSIC, for Colombia and a specific permit was issued for this purpose on the 28-06-2011 by the Conservation Office of the Ascension Island Government (website) to PvW University of Aberdeen UK. Dr. Andrea Phillott obtained DNA sequences of fungal samples from Australia. These fungal samples were obtained from successfully hatched or dead eggs following regulations of Queensland Turtle Research, Queensland Parks and Wildlife Service, Australia.

When samples of eggshells required to be exported these were done under the following permits: CITES 10/08, 1/09, 003/VS and 08CR000006/SJ. Studies carried out on nests in Cape Verde were performed following protocols of the Directive of the Bioethics Committee of the Consejo Superior de Investigaciones Cientificas, CSIC (www.ebd.csic.es/bioetica/index.html) by agreement to Cape Verde Regulations (JMSR, AM, EAP, JDU). None of the experiments involved sacrificing animals and, therefore we did not require a specific approval from any institutional animal research ethics committee.

### Disease survey, collection of diseased eggs, and fungal isolations

Sea turtle eggshells with macroscopic signs of *Fusarium* infection [9] from all stages of embryonic development (Figure 2) were collected from six of the major sea turtle-nesting regions in the Atlantic, Indian and Pacific Oceans, and the Caribbean Sea during the period of 2005 to 2012. Samples were collected from the following species and locations: *Chelonia mydas* (Raine Island, Australia; Isla de la Plata at Machalilla National Park, Ecuador; and Ascension Island), *Caretta caretta* (Boa Vista Island, Cape Verde), *Eretmochelys imbricata* and *Lepidochelys olivacea* (La Playita, Machalilla National Park), *Dermochelys coriacea* (La Playona, Colombia and Pacuare Nature Reserve, Costa Rica), and *Natator depressus* (Crab Island, Australia) (Figure 1 and Table S1). In order to elucidate the source and the progress of the *Fusarium* disease, additional samples were also collected, *i.e.*, eggs with signs of *Fusarium* infection at early stages of incubation, eggs not showing signs of fungal disease at late stages of incubation (Figure 2), and also eggs directly from the ovipositor of the nesting female and cloaca mucus, from the species *E. imbricata* at Machalilla National Park, Ecuador. Eggs with embryos at stage 1 to 4 of development were considered at early stage of incubation, and those at 5 to 9 were considered late stage [45]. Samples from living eggs were taken without sacrificing the embryos by sampling the surface with sterile swabs. Samples from embryos were only taken when these were dead. The determination of whether the embryo was alive or dead was based on the presence of a persistent white spot on the upper surface of the eggshell. Thus, the embryo was considered alive when the white spot was observed [45], [46].

Selected fragments of the eggshells, fungal swabs and dilution series of cloaca mucus were placed onto PG-1 (peptone glucose agar) with ampicillin (100 mg/l) [47]. The resulting fungal colonies were sub-cultured to produce axenic cultures. The strains were deposited in the culture collection of the Real Jardín Botánico-CSIC, Madrid, Spain.

### Phenotypic and molecular characterization of *Fusarium* isolates from sea turtle eggs

The obtained sea turtle isolates (STI) were morphologically compared to isolates of *F. solani* non-sea turtle isolates (NSTI), *i.e.*, from other environments and hosts, acquired from the Fungal Genetics Stock Center MO, and Universities of Rovira i Virgili and Salamanca of Spain (Table S1). Stock isolates were cultured on potato dextrose agar (PDA, Difco, MI) at 23°C. Cultures in 90 mm Petri dishes were used to study the colony color [48] and microscopic morphology [49]. Conidia, chlamydospores and mycelia were observed using an Olympus BX-51 compound microscope (Olympus Optical, Tokyo, Japan). Species identification was carried out following the manuals of Booth [25] and Nelson [26] for *Fusarium* spp. Light micrographs were captured using a Micropublisher 5.0 digital camera (Qimaging, Burnaby, BC, Canada) and the software SYNCROSCOPY-AUTOMONTAGE (Microbiology International Inc., Frederick, MD) as described in Diéguez-Uribeondo [50] (Figure S3).

In order to molecularly characterize the isolates, DNA was extracted from pure cultures using the DNA Easy PlantMini Kit (Qiagen, Valencia, CA). The primer pairs ITS5/ITS4 [51], LROR/LR6 [52], [53] and fRPB2-7cf/fRPB2-11aR [54] and were used to study the diversity of the internal transcribed spacer (ITS) and the nuclear large subunit (LSU) of the nuclear ribosomal DNA (nrDNA), and the second large subunit of the RNA polymerase II (*RPB2*) gene. The amplification products were sequenced by MACROGEN (Inc. Seoul, Korea). Sequencher (Gene Codes Corporation, Ann Arbor, MI) was used to identify the consensus sequence from the two strands of the ITS nrDNA, LSU nrDNA and *RPB2* of each isolate. For initial identification of the isolates the ITS nrDNA sequences were compared with those of the National Center of Biotechnology Information (NCBI) nucleotide databases using the Nucleotide BLASTN tool. The ITS nrDNA region was characterized for all the *F. solani* isolates obtained from sea turtles and other hosts (Figure S1). LSU nrDNA and *RPB2* were obtained from 38 STI, and 8 NSTI (Table S1) and analyzed with additional GenBank sequences of these DNA regions from 60 isolates of *Fusarium* spp. (Table S2).

### Phylogenetic analyses

Combined analyses of three loci were conducted to identify phylogenetic species of the sea turtle pathogenic isolates, within the FSSC. Sequences of the three loci, ITS nrDNA, LSU nrDNA and *RPB2* gene were used for phylogenetic analyses. GenBank sequences of *Fusarium* turtle egg isolates (Table S1) and selected homologous GenBank sequences of *Fusarium* spp. from other hosts and environments were included (Table S2). The program Se-Al 2.0a11 Carbon [55] was used for manual alignment of the sequences. Maximum parsimony analyses (MP) [56] was inferred using the heuristic search option in PAUP*4.0b10. Nonparametric bootstrap support (BS) [57] for each clade was tested based on 10,000 replicates, using the fast-step option. The consistency index, CI [58] and retention index, RI [59] were calculated. A second analysis was conducted using a Bayesian approach with Mr. Bayes 3.1 [60]. Posterior probabilities (PP) [61], [62] were approximated by sampling trees using a Markov Chain Montecarlo (MCMC) method. The analysis was performed assuming the general time reversible model [63] including estimation of invariant sites and assuming a discrete gamma distribution with six categories (GTR+I+G). The model was obtained with Mr Modeltest 2.3 [64]. A run with 2,000,000 generations, and employing 12 simultaneous chains, was executed. Every 100^th^ tree was saved into a file. The initial 1,000 trees obtained were discarded and a 50% majority-rule consensus tree was obtained from the last 19,000 trees sampled. A combination of both posterior probabilities and bootstrap proportion was used to assess confidence for a specific node [65]. The analyses were conducted on the sequences of the three loci as individual and combined datasets. Phylogenetic trees were edited with TreeView [66]. The alignment matrix and consensus tree obtained from the combined Bayesian analysis are available at TreeBase (http://purl.org/phylo/treebase/phylows/study/TB2:S13912).

### *Fusarium* disease incidence and embryonic mortality rate

In order to study the relationship between *Fusarium* disease incidence and embryo mortality rate, the number of eggs that showed signs of the disease was compared with the egg mortality rate in field conditions. *Caretta caretta* nests (*n* = 102) were collected from different nesting beaches in Boa Vista Island and relocated into a hatchery at Ervatao beach, Cape Verde in 2011. All nests were excavated 60 days after the beginning of incubation. The numbers of hatched and unhatched eggs (with and without signs of embryonic development) was recorded. The number of unhatched eggs with signs embryonic development and fungal growth was also recorded. Disease incidence per nest was calculated as the number of dead eggs with internal and external signs of *Fusarium* infection as a proportion of the total clutch size. Mortality rate per nest was calculated as the number of unhatched eggs as a proportion of the total clutch size. A regression analysis of *F. solani* disease incidence and mortality rate, and scatterplot of this analysis, with the statistics R^2^, correlation, *P* values and regression equation, was obtained using the program Statistica version 6.0 (StatSoft, Inc. US) (Figure S4).

### Temperature relationship for mycelial growth and optimal growth temperature

Selected isolates representing *F. solani* STI and NSTI from each phylogenetic subclade and location were tested, *i.e.*, 12 STI from subclade A (001B, 006, 033, 043, 050, 078, 082, 131, 141, 209, 217 and 220FUS), 12 STI from subclade C (001C, 029, 030, 035, 047, 056, 057, 092, 110, 117, 228 and 230FUS) and 12 NSTI from subclade B (133, 136, 137, 138, 188, 189, 190, 191, 192, 193, 194 and 195FUS) (See Table S1). These relationships were determined by measuring the mycelia growth rate on PDA cultures incubated at 10, 21, 25, 27, 29, 32 and 37°C in daylight. Agar plugs (5 mm in diameter) of the isolates were inoculated in 90 mm Petri dishes. Each isolate was cultured at every temperature in triplicate. Mycelia growth per day was calculated by measuring the radius (mm) of the colony every 24 h from 1 to 5 days after inoculation [24]. The optimal growth temperature, OGT, was estimated by obtaining the best-fit equation for the temperature-radial growth relationship using Microsoft Excel, and calculating the maximum value of the best-fit equation for each isolate. The OGT for each isolate was assessed as the mean value from three repetitions. The mean OGT was also calculated for each phylogenetic subclade, and was estimated as the average from the three replicates. The differences in OGT among subclades A, B and C (Figure 4) were statistically analyzed by analysis of variance (One-way ANOVA) and means were compared with a *post hoc* Tukey test provided in the statistical algorithms of Statistica version 6.0 (StatSoft, Inc. US).

### Challenge inoculation experiments

In order to evaluate the host range of the *F. falciforme* and *F. keratoplasticum* STI, tubers of *Solanum tuberosum* were challenged under controlled conditions. Selected isolates from subclade A (043, 050 and 033FUS) and C (057, 031 and 029FUS) were tested (See Table S1). Previous to the inoculation, the tubers were rinsed with tap water and sterilized by immersion in 10% sodium hypochlorite for 5 min [67], then rinsed 3 times with sterile distilled water and air-dried overnight. Nine wounds were induced on the tubers by introducing the tip of sterile tweezers (1 cm) in them. Each wound was inoculated with 500 µl of conidial suspensions (2×10^6^ conidia/ml). The controls were inoculated with sterile tap water. The inoculated tubers were maintained in plastic containers, separated by treatments, and incubated at 25°C. After 2 weeks of incubation the tubers were transversally cut and evaluated for signs of *Fusarium* dry rot disease, i.e., dark depressions in the surface of the tubers and necrotic dry tissue along the wounds [68]. Four tubers were inoculated per treatment. The experiment was repeated twice.

### Evaluation of the effect of tidal inundation and substrate composition on sea turtle egg mortality rate and *Fusarium* disease incidence

Two environmental conditions, *i.e.*, clay/silt composition of the substrate and tidal inundation, were tested for their influence on disease development in nests of sea turtle *C. caretta*. A total of 29 nests of *C. caretta* differentially exposed to these conditions were studied during the 2011-nesting season in Boa Vista Island. Sand composition was determined based on granulometric studies of selected nesting regions [42]. Inundation was assessed based on daily monitoring of the nests. Thus, three categories of nests were studied: sandy nests not exposed to inundations (*n* = 13); sandy nests exposed to inundations (*n* = 8); and, sandy nests that possess clay and/or silt substrates (*n* = 8). All nests were physically examined from the first day of incubation until the first hatchling emerged and exhumed 60 days after the beginning of incubation. Disease incidence was determined for 5 nests per category. Disease incidence and mortality rate per nest were calculated as previously described. *Fusarium* disease incidence and mortality rate per nest category were calculated as the mean value of all the nests monitored within each category (Figure 5). Differences on disease incidence and mortality rate among types of beaches were statistically analyzed by analyses of variance (One-way ANOVA) and means were compared by *post hoc* Tukey test provided in the statistical algorithms of Statistica version 6.0 (StatSoft, Inc. US).

## Supporting Information

Figure S1 **Out-group-rooted cladogram of the ITS nrDNA region.** One of the most parsimonious three inferred from the sequence ITS nrDNA data for 119 sea turtle isolates and 62 non sea turtle isolates within the *Fusarium solani* species complex. The numbers on the internodes indicate the posterior probability (PP) and the bootstrap values (BS) of the parsimony and Bayesian analysis when (PP≥0.95 and BS≥70%). Consistency index (CI) = 0.27. Retention index (RI) = 0.58. Eight plant host-specific formae speciales of the polytypic morphospecies *F. solani* are indicated with the f. sp. prefix.
(TIF)

Figure S2 **Bootstrap multilocus out-group-rooted cladogram.** Cladogram inferred from the combined DNA sequence data from tree loci (ITS nrDNA, LSU nrDNA and RPB2) for 38 sea turtle and 62 non sea turtle isolates. Numbers on the internodes indicate the posterior probability (PP) and the bootstrap values (BS) of the parsimony and Bayesian analysis when PP≥0.95 and BS≥70%. Consistency index (CI) = 0.53. Retention index (RI) = 0.86. Eight plant host-specific formae speciales of the polytypic morphospecies *F. solani* are indicated with the f.sp. prefix. A solid asterisk to the right of an NRRL number identifies the *Fusarium falciforme* isolates. A solid square to the right of an NRRL number identifies the *Fusarium keratoplasticum* isolates.
(TIF)

Figure S3 **Characteristic morphology of** ***Fusarium*** **spp. pathogenic to sea turtle eggs.** (A–C) *F. keratoplasticum* asexual structures: A) Septate fusoid microconidia (m) and hyphae (h). B) Branched monophialide (mp) bearing microconidia. C) Globose, smooth walled chlamydospores (ch), terminal in hyphae. D–F) *F. falciforme* asexual structures: D) Aseptate and septate, ellipsoidal to reniform microconidia. E) Branched monophialides bearing microconidia. F) Globose, smooth walled chlamydospores, intercalary in hyphae. Scale bar = 5 µm.
(TIF)

Figure S4 **Regression of** ***Fusarium*** **disease incidence on the embryonic mortality rate.** The *Fusarium*-disease incidence and embryonic mortality rate were obtained from nests of the sea turtle species *Caretta caretta* in Boa Vista, Cape Verde. Circles represent the data for each nest. Line indicates the best-fit regression (y = 11.26+0.90 * x, r^2^ = 0.87, *p*<0.01) (n = 102).
(TIF)

Figure S5 **Tubers of** ***Solanum tuberosum*** **challenged with sea turtle isolates from two species of** ***Fusarium*** **.** The two *Fusarium* species belong to the *Fusarium solani* species complex. (A–D) Tuber not inoculated with *Fusarium* spp. Tubers with typical superficial and internal symptoms of the *Fusarium* dry rot infection: (B–E) tuber inoculated with *F. falciforme* and (C–F) tuber inoculated with *F. keratoplasticum*. Arrows indicates the location of the wounds and inoculation points.
(TIF)

Table S1 Isolates of the Fusarium solani species complex collected from sea turtle eggs, plants, or environmental samples.
(DOCX)

Table S2 GenBank sequences of the DNA regions: ITS nrDNA, 28S nrDNA, and RPB2 of the isolates of the Fusarium solani species complex included in the phylogenetic analyses.
(DOCX)
