# Supplementary figures and images for: Global Distribution of Two Fungal Pathogens Threatening Endangered Sea Turtles
Source: PLoS One. 2014 Jan 21;9(1):e85853. doi: 10.1371/journal.pone.0085853 (PMC3897526; doi:10.1371/journal.pone.0085853)

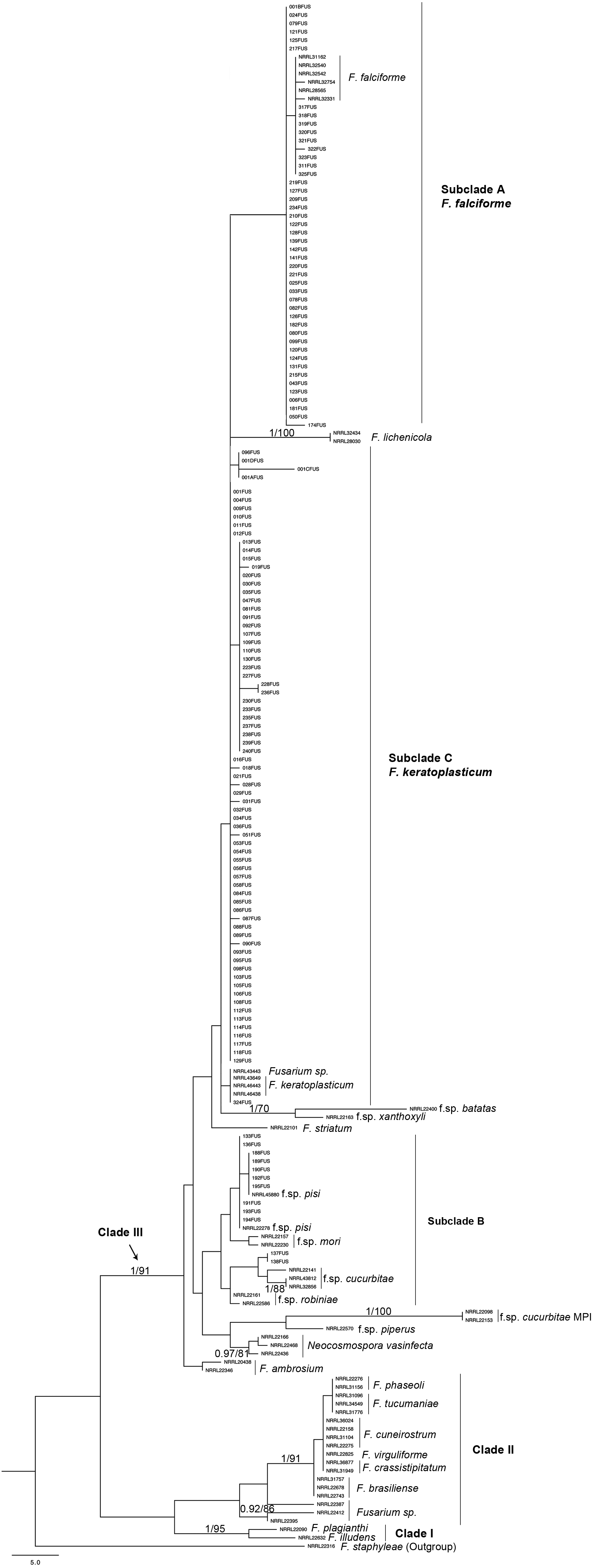

Supplement: Figure S1 — Out-group-rooted cladogram of the ITS nrDNA region. One of the most parsimonious three inferred from the sequence ITS nrDNA data for 119 sea turtle isolates and 62 non sea turtle isolates within the Fusarium solani species complex. The numbers on the internodes indicate the posterior probability (PP) and the bootstrap values (BS) of the parsimony and Bayesian analysis when (PP≥0.95 and BS≥70%). Consistency index (CI) = 0.27. Retention index (RI) = 0.58. Eight plant host-specific formae speciales of the polytypic morphospecies F. solani are indicated with the f. sp. prefix. (TIF) [file pone.0085853.s001.tif]

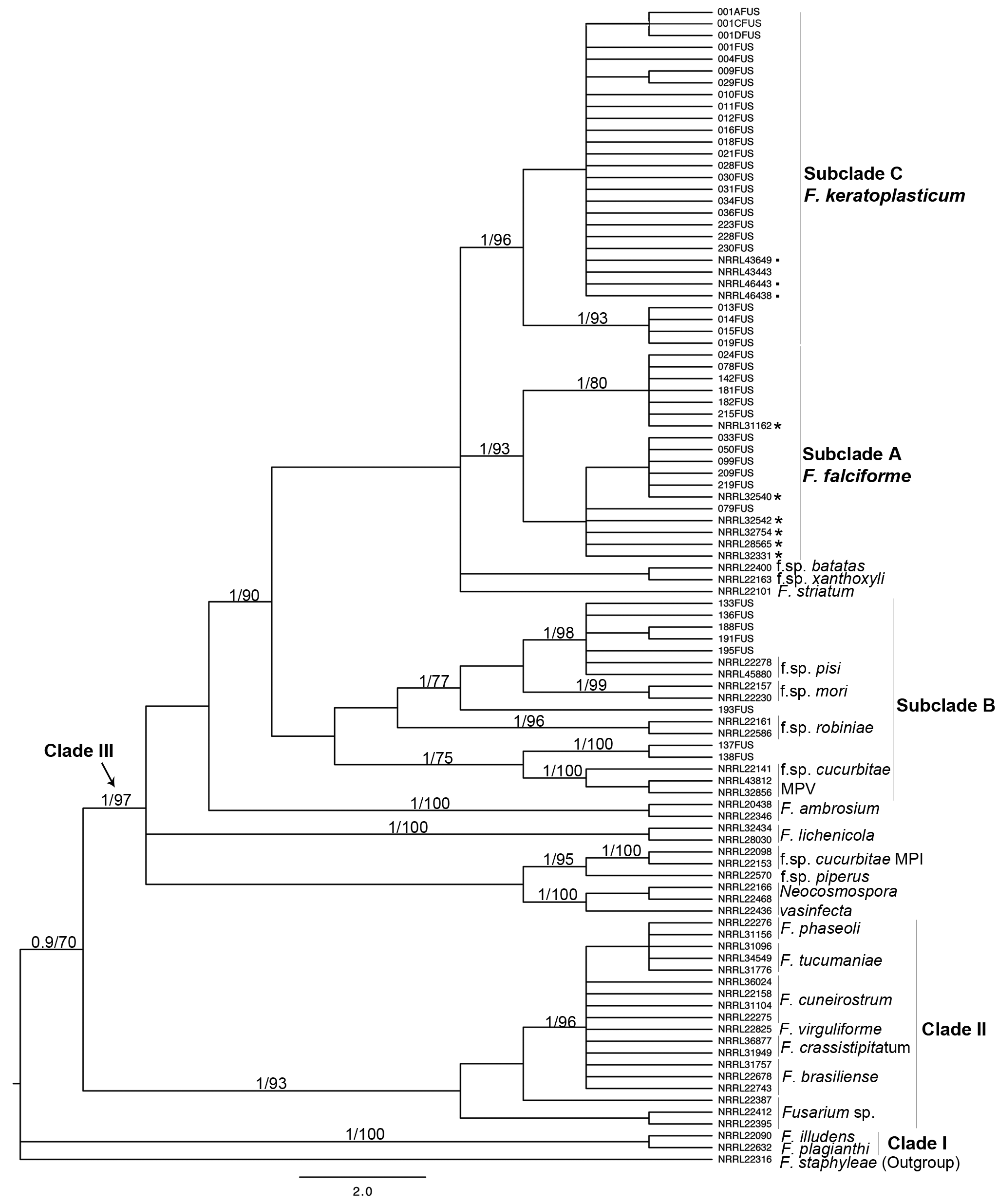

Supplement: Figure S2 — Bootstrap multilocus out-group-rooted cladogram. Cladogram inferred from the combined DNA sequence data from tree loci (ITS nrDNA, LSU nrDNA and RPB2) for 38 sea turtle and 62 non sea turtle isolates. Numbers on the internodes indicate the posterior probability (PP) and the bootstrap values (BS) of the parsimony and Bayesian analysis when PP≥0.95 and BS≥70%. Consistency index (CI) = 0.53. Retention index (RI) = 0.86. Eight plant host-specific formae speciales of the polytypic morphospecies F. solani are indicated with the f.sp. prefix. A solid asterisk to the right of an NRRL number identifies the Fusarium falciforme isolates. A solid square to the right of an NRRL number identifies the Fusarium keratoplasticum isolates. (TIF) [file pone.0085853.s002.tif]

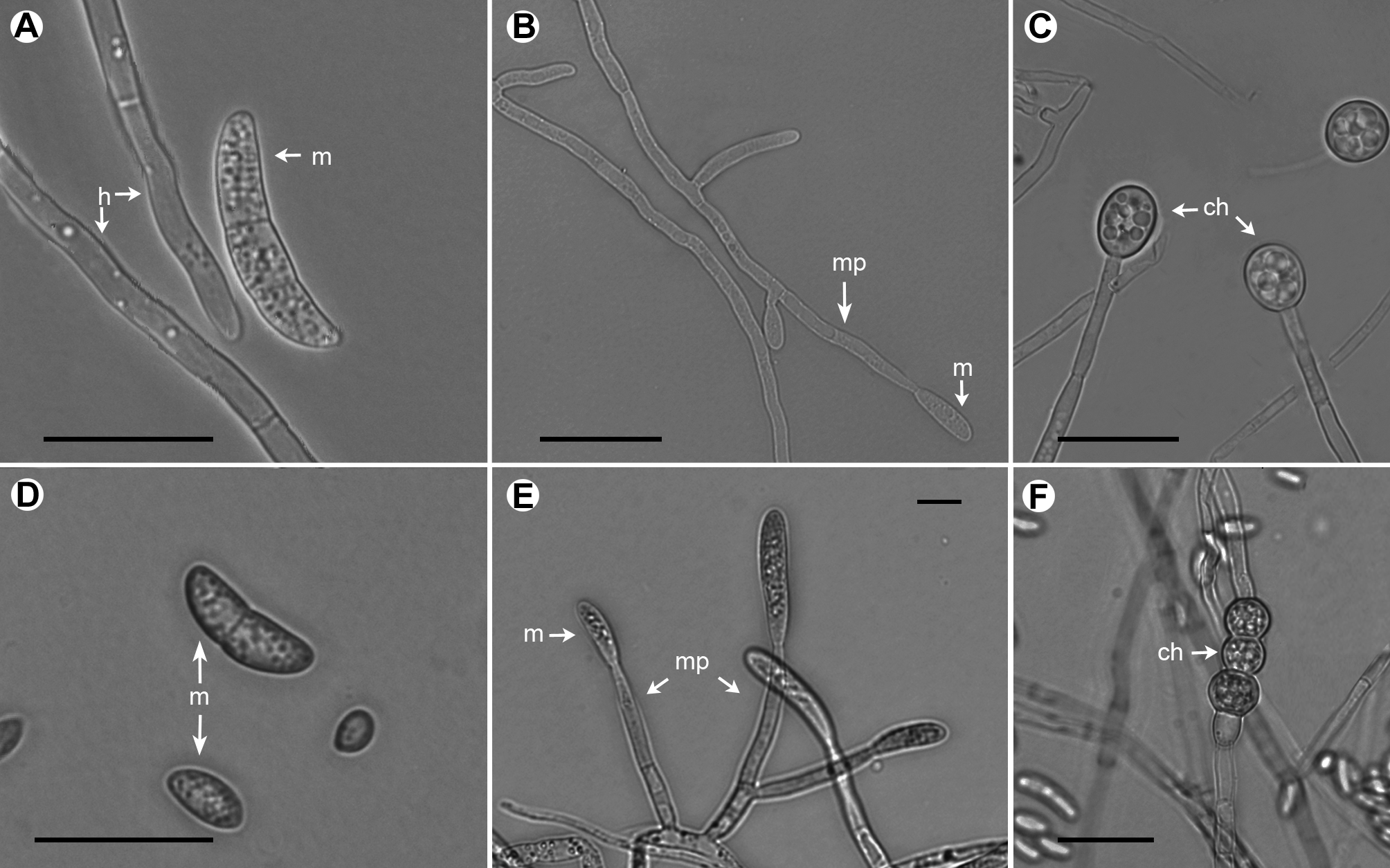

Supplement: Figure S3 — Characteristic morphology of Fusarium spp. pathogenic to sea turtle eggs. (A–C) F. keratoplasticum asexual structures: A) Septate fusoid microconidia (m) and hyphae (h). B) Branched monophialide (mp) bearing microconidia. C) Globose, smooth walled chlamydospores (ch), terminal in hyphae. D–F) F. falciforme asexual structures: D) Aseptate and septate, ellipsoidal to reniform microconidia. E) Branched monophialides bearing microconidia. F) Globose, smooth walled chlamydospores, intercalary in hyphae. Scale bar = 5 µm. (TIF) [file pone.0085853.s003.tif]

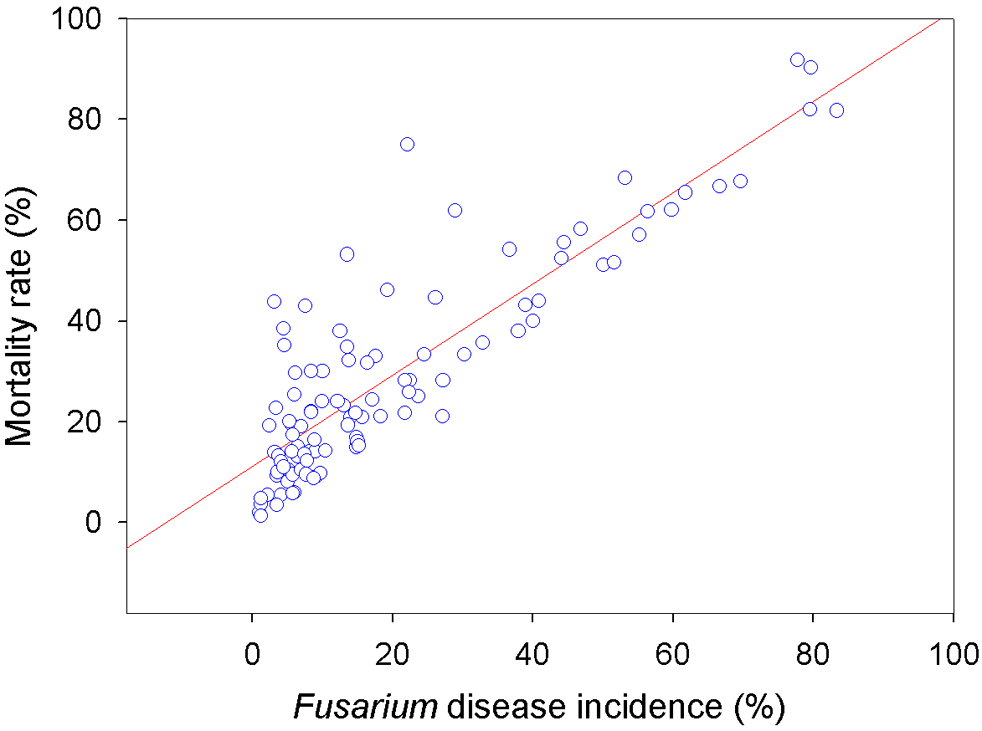

Supplement: Figure S4 — Regression of Fusarium disease incidence on the embryonic mortality rate. The Fusarium-disease incidence and embryonic mortality rate were obtained from nests of the sea turtle species Caretta caretta in Boa Vista, Cape Verde. Circles represent the data for each nest. Line indicates the best-fit regression (y = 11.26+0.90 * x, r2 = 0.87, p<0.01) (n = 102). (TIF) [file pone.0085853.s004.tif]

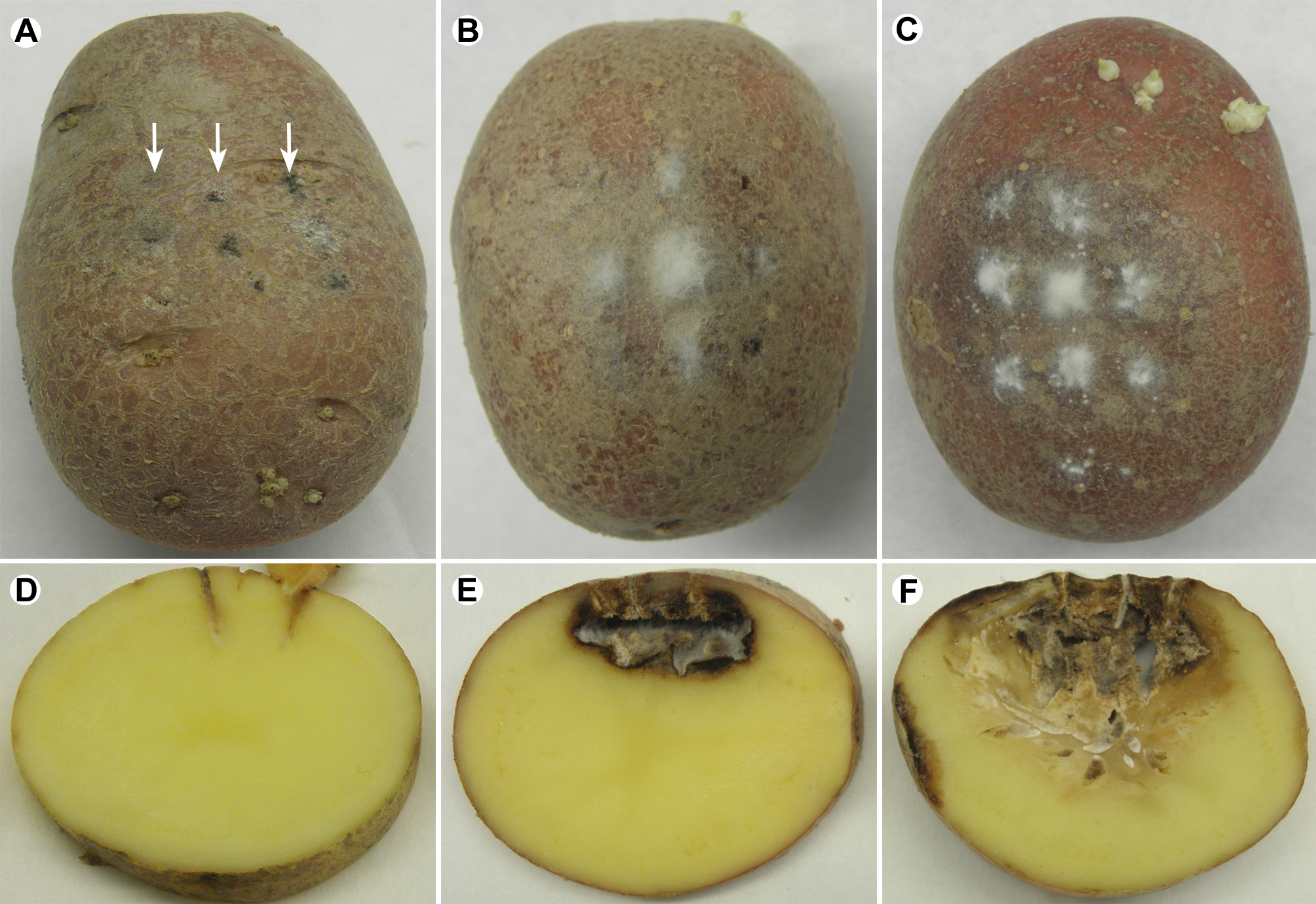

Supplement: Figure S5 — Tubers of Solanum tuberosum challenged with sea turtle isolates from two species of Fusarium . The two Fusarium species belong to the Fusarium solani species complex. (A–D) Tuber not inoculated with Fusarium spp. Tubers with typical superficial and internal symptoms of the Fusarium dry rot infection: (B–E) tuber inoculated with F. falciforme and (C–F) tuber inoculated with F. keratoplasticum. Arrows indicates the location of the wounds and inoculation points. (TIF) [file pone.0085853.s005.tif]
