# Supplementary material for: Global Distribution of Two Fungal Pathogens Threatening Endangered Sea Turtles
Source: PLoS One. 2014 Jan 21;9(1):e85853. doi: 10.1371/journal.pone.0085853 (PMC3897526; doi:10.1371/journal.pone.0085853)
Supplement: Table S1 — Isolates of the Fusarium solani species complex collected from sea turtle eggs, plants, or environmental samples. (DOCX) [file pone.0085853.s006.docx]

**Table S1.** *Fusarium solani* isolates collected from sea turtle eggs, plants, and environmental samples.

|  |  |  |  |  | GenBank Accession^b^ |  |  |  |
| --- | --- | --- | --- | --- | --- | --- | --- | --- |
| Clade | Subclade | Strain^a^ | Host | ITS nrDNA | 28S nrDNA | *RPB2* | Source^c*^ | Origin |
| III | A | 001B FUS | *Caretta caretta* | FR691777 | - | - | SIS | Cape Verde |
| III | A | 006 FUS | *Dermochelys coriacea* | KC573924 | - | - | SIS | Colombia |
| III | A | 024 FUS | *Eretmochelys imbricata* | KC573925 | KC573882 | KC574029 | SIS | Ecuador |
| III | A | 025 FUS | *Eretmochelys imbricata* | KC573926 | - | - | SIS | Ecuador |
| III | A | 033 FUS | *Chelonia mydas* | KC573932 | KC573883 | KC574030 | SIS | Ecuador |
| III | A | 043 FUS | *Chelonia mydas* | KC573936 | - | - | SIS | Ecuador |
| III | A | 050 FUS | *Caretta caretta* | FR691776 | JN939552 | KC574031 | EIS | Cape Verde |
| zIII | A | 078 FUS | *Caretta caretta* | KC573938 | KC573884 | KC574032 | IE | Cape Verde |
| III | A | 079 FUS | *Caretta caretta* | KC573939 | KC573885 | KC574033 | SIS | Cape Verde |
| III | A | 080 FUS | *Caretta caretta* | KC573940 | - | - | SIS | Cape Verde |
| III | A | 082 FUS | *Caretta caretta* | KC573942 | - | - | IE | Cape Verde |
| III | A | 099 FUS | *Caretta caretta* | KC573956 | KC573886 | KC574034 | IE | Cape Verde |
| III | A | 120 FUS | *Caretta caretta* | KC573970 | - | - | SIS | Cape Verde |
| III | A | 121 FUS | *Caretta caretta* | KC573971 | - | - | SIS | Cape Verde |
| III | A | 122 FUS | *Caretta caretta* | KC573972 | - | - | SIS | Cape Verde |
| III | A | 123 FUS | *Caretta caretta* | KC573973 | - | - | SIS | Cape Verde |
| III | A | 124 FUS | *Caretta caretta* | KC573974 | - | - | SIS | Cape Verde |
| III | A | 125 FUS | *Caretta caretta* | KC573975 | - | - | SIS | Cape Verde |
| III | A | 126 FUS | *Caretta caretta* | KC573976 | - | - | SIS | Cape Verde |
| III | A | 127 FUS | *Caretta caretta* | KC573977 | - | - | SIS | Cape Verde |
| III | A | 128 FUS | *Caretta caretta* | KC573978 | - | - | IE | Cape Verde |
| III | A | 131 FUS | *Caretta caretta* | KC573981 | - | - | IE | Cape Verde |
| III | A | 139 FUS | *Chelonia mydas* | KC573986 | - | - | SIS | Ecuador |
| III | A | 141 FUS | *Chelonia mydas* | KC573988 | - | - | SIS | Ecuador |
|  |  |  |  |  | GenBank Accession^b^ |  |  |  |
| Clade | Subclade | Strain^a^ | Host | ITS nrDNA | 28S nrDNA | *RPB2* | Source^c*^ | Origin |
| III | A | 142 FUS | *Chelonia mydas* | KC573987 | KC573887 | KC574035 | SIS | Ecuador |
| III | A | 174 FUS | *Chelonia mydas* | KC573989 | - | - | SIS | Australia |
| III | A | 181 FUS | *Natator depressus* | KC573990 | KC573888 | KC574036 | SIS | Australia |
| III | A | 182 FUS | *Natator depressus* | KC573991 | KC573889 | KC574037 | SIS | Australia |
| III | A | 209 FUS | *Lepidochelys olivacea* | KC574000 | KC573890 | KC574038 | SIS | Ecuador |
| III | A | 210 FUS | *Chelonia mydas* | KC574001 | - | - | SIS | Ecuador |
| III | A | 215 FUS | *Lepidochelys olivacea* | KC574002 | KC573891 | KC574039 | SIS | Ecuador |
| III | A | 217 FUS | *Lepidochelys olivacea* | KC574003 | - | - | SIS | Ecuador |
| III | A | 219 FUS | *Lepidochelys olivacea* | KC574004 | KC573892 | KC574040 | SIS | Ecuador |
| III | A | 220 FUS | *Eretmochelys imbricata* | KC574005 | - | - | SIS | Ecuador |
| III | A | 221 FUS | *Eretmochelys imbricata* | KC574006 | - | - | SIS | Ecuador |
| III | A | 234 FUS | *Chelonia mydas* | KC574012 | - | - | SIS | Ascencion Island |
| III | A | 311 FUS | *Chelonia mydas* | KC574026 | - | - | SIS | Australia |
| III | A | 317 FUS | *Chelonia mydas* | KC574019 | - | - | SIS | Australia |
| III | A | 318 FUS | *Natator depressus* | KC574020 | - | - | SIS | Australia |
| III | A | 319 FUS | *Natator depressus* | KC574021 | - | - | SIS | Australia |
| III | A | 320 FUS | *Natator depressus* | KC574022 | - | - | SIS | Australia |
| III | A | 321 FUS | *Natator depressus* | KC574023 | - | - | SIS | Australia |
| III | A | 322 FUS | *Natator depressus* | KC574024 | - | - | SIS | Australia |
| III | A | 323 FUS | *Natator depressus* | KC574025 | - | - | SIS | Australia |
| III | A | 325 FUS | *Dermochelys coriacea* | KC574028 | - | - | AE* | Costa Rica |
| III | A | 336FUS | *Eretmochelys imbricata* | KF179256 | - | - | EIS* | Ecuador |
| III | A | 337FUS | *Eretmochelys imbricata* | KF179257 | - | - | EIS* | Ecuador |
| III | A | 338FUS | *Eretmochelys imbricata* | KF179258 | - | - | EIS* | Ecuador |
| III | A | 339FUS | *Eretmochelys imbricata* | KF179259 | - | - | EIS* | Ecuador |
|  |  |  |  |  | GenBank Accession^b^ |  |  |  |
| Clade | Subclade | Strain^a^ | Host | ITS nrDNA | 28S nrDNA | *RPB2* | Source^c*^ | Origin |
| III | A | 340FUS | *Eretmochelys imbricata* | KF179260 | - | - | EIS* | Ecuador |
| III | A | 341FUS | *Eretmochelys imbricata* | KF179261 | - | - | EIS* | Ecuador |
| III | A | 370FUS | *Eretmochelys imbricata* | KF683304 | - | - | AE | Ecuador |
| III | A | 371FUS | *Eretmochelys imbricata* | KF683305 | - | - | AE | Ecuador |
| III | A | 372FUS | *Eretmochelys imbricata* | KF683306 | - | - | AE | Ecuador |
| III | A | 373FUS | *Eretmochelys imbricata* | KF683307 | - | - | AE | Ecuador |
| III | B | 133 FUS | *Lupinus albus* | KC573982 | KC573893 | KC574041 | IP | Spain |
| III | B | 136 FUS | *Lupinus albus* | KC573983 | KC573894 | KC574042 | IP | Spain |
| III | B | 137 FUS | Aquarium | KC573984 | KC573895 | KC574043 | Aquarium | Spain |
| III | B | 138 FUS | Aquarium | KC573985 | KC573896 | KC574044 | Aquarium | Spain |
| III | B | 188 FUS | *Pisum sativum* | KC573992 | KC573897 | KC574045 | IP | unknown |
| III | B | 189 FUS | *Pisum sativum* | KC573993 | - | - | IP | unknown |
| III | B | 190 FUS | *Pisum sativum* | KC573994 | - | - | IP | unknown |
| III | B | 191 FUS | *Pisum sativum* | KC573995 | KC573898 | KC574046 | IP | unknown |
| III | B | 192 FUS | *Pisum sativum* | KC573996 | - | - | IP | unknown |
| III | B | 193 FUS | *Pisum sativum* | KC573997 | KC573899 | KC574047 | IP | unknown |
| III | B | 194 FUS | *Pisum sativum* | KC573998 | - | - | IP | unknown |
| III | B | 195 FUS | *Pisum sativum* | KC573999 | KC573900 | KC574048 | IP | US |
| III | C | 001C FUS | *Caretta caretta* | FR691754 | KC594706 | KC594707 | IE | Cape Verde |
| III | C | 001A FUS | *Caretta caretta* | FR691753 | JN939570 | KC574049 | IE | Cape Verde |
| III | C | 001D FUS | *Caretta caretta* | FR691755 | JN939553 | KC574050 | SIS | Cape Verde |
| III | C | 001 FUS | *Dermochelys coriacea* | KC573923 | KC573901 | KC574051 | SIS | Colombia |
| III | C | 004 FUS | *Caretta caretta* | FR691856 | KC573902 | KC574052 | SIS | Cape Verde |
| III | C | 009 FUS | *Caretta caretta* | FR691760 | KC573903 | KC574053 | SIS | Cape Verde |
| III | C | 010 FUS | *Caretta caretta* | FR691761 | KC573904 | KC574054 | IE | Cape Verde |
|  |  |  |  |  | GenBank Accession^b^ |  |  |  |
| Clade | Subclade | Strain^a^ | Host | ITS nrDNA | 28S nrDNA | *RPB2* | Source^c*^ | Origin |
| III | C | 011 FUS | *Caretta caretta* | FR691762 | KC573905 | KC574055 | IE | Cape Verde |
| III | C | 012 FUS | *Caretta caretta* | FR691763 | KC573906 | KC574056 | SIS | Cape Verde |
| III | C | 013 FUS | *Caretta caretta* | FR691764 | KC573907 | KC574057 | SIS | Cape Verde |
| III | C | 014 FUS | *Caretta caretta* | FR691757 | KC573908 | KC574058 | SIS | Cape Verde |
| III | C | 015 FUS | *Caretta caretta* | FR691759 | KC573909 | KC574059 | SIS | Cape Verde |
| III | C | 016 FUS | *Caretta caretta* | FR691758 | KC573910 | KC574060 | SIS | Cape Verde |
| III | C | 018 FUS | *Caretta caretta* | FR691765 | KC573911 | KC574061 | SIS | Cape Verde |
| III | C | 019 FUS | *Caretta caretta* | FR691766 | KC573912 | KC574062 | SIS | Cape Verde |
| III | C | 020 FUS | *Caretta caretta* | FR691767 | - | - | SIS | Cape Verde |
| III | C | 021 FUS | *Caretta caretta* | FR691768 | KC573913 | KC574063 | IE | Cape Verde |
| III | C | 028 FUS | *Chelonia mydas* | KC573927 | KC573914 | KC574064 | SIS | Ecuador |
| III | C | 029 FUS | *Eretmochelys imbricata* | KC573928 | KC573915 | KC574065 | SIS | Ecuador |
| III | C | 030 FUS | *Eretmochelys imbricata* | KC573929 | KC573916 | KC574066 | SIS | Ecuador |
| III | C | 031 FUS | *Chelonia mydas* | KC573930 | KC573917 | KC574067 | SIS | Ecuador |
| III | C | 032 FUS | *Chelonia mydas* | KC573931 | - | - | SIS | Ecuador |
| III | C | 034 FUS | *Chelonia mydas* | KC573933 | KC573918 | KC574068 | SIS | Ecuador |
| III | C | 035 FUS | *Eretmochelys imbricata* | KC573934 | - | - | SIS | Ecuador |
| III | C | 036 FUS | *Chelonia mydas* | KC573935 | KC573919 | KC574069 | SIS | Ecuador |
| III | C | 047 FUS | *Chelonia mydas* | KC573937 | - | - | SIS | Ecuador |
| III | C | 051 FUS | *Caretta caretta* | FR691769 | - | - | EIS | Ecuador |
| III | C | 053 FUS | *Caretta caretta* | FR691770 | - | - | IE | Cape Verde |
| III | C | 054 FUS | *Caretta caretta* | FR691771 | - | - | IE | Cape Verde |
| III | C | 055 FUS | *Caretta caretta* | FR691772 | - | - | SIS | Cape Verde |
| III | C | 056 FUS | *Caretta caretta* | FR691773 | - | - | IE | Cape Verde |
| III | C | 057 FUS | *Caretta caretta* | FR691774 | - | - | EIS | Cape Verde |
|  |  |  |  |  | | |  |  |
|  |  |  |  |  | | |  |  |
|  |  |  |  |  | GenBank Accession^b^ |  |  |  |
| Clade | Subclade | Strain^a^ | Host | ITS nrDNA | 28S nrDNA | *RPB2* | Source^c*^ | Origin |
| III | C | 058 FUS | *Caretta caretta* | FR691775 | - | - | EIS | Cape Verde |
| III | C | 081 FUS | *Caretta caretta* | KC573941 | - | - | SIS | Cape Verde |
| III | C | 084 FUS | *Caretta caretta* | KC573942 | - | - | SIS | Cape Verde |
| III | C | 085 FUS | *Caretta caretta* | KC573944 | - | - | SIS | Cape Verde |
| III | C | 086 FUS | *Caretta caretta* | KC573945 | - | - | SIS | Cape Verde |
| III | C | 087 FUS | *Caretta caretta* | KC573946 | - | - | SIS | Cape Verde |
| III | C | 088 FUS | *Caretta caretta* | KC573947 | - | - | SIS | Cape Verde |
| III | C | 089 FUS | *Caretta caretta* | KC573948 | - | - | SIS | Cape Verde |
| III | C | 090 FUS | *Caretta caretta* | KC573949 | - | - | SIS | Cape Verde |
| III | C | 091 FUS | *Caretta caretta* | KC573950 | - | - | SIS | Cape Verde |
| III | C | 092 FUS | *Caretta caretta* | KC573951 | - | - | SIS | Cape Verde |
| III | C | 093 FUS | *Caretta caretta* | KC573952 | - | - | SIS | Cape Verde |
| III | C | 095 FUS | *Caretta caretta* | KC573953 | - | - | SIS | Cape Verde |
| III | C | 096 FUS | *Caretta caretta* | KC573954 | - | - | SIS | Cape Verde |
| III | C | 098 FUS | *Caretta caretta* | KC573955 | - | - | SIS | Cape Verde |
| III | C | 103 FUS | *Caretta caretta* | KC573957 | - | - | SIS | Cape Verde |
| III | C | 105 FUS | *Caretta caretta* | KC573958 | - | - | SIS | Cape Verde |
| III | C | 106 FUS | *Caretta caretta* | KC573959 | - | - | SIS | Cape Verde |
| III | C | 107 FUS | *Caretta caretta* | KC573960 | - | - | SIS | Cape Verde |
| III | C | 108 FUS | *Caretta caretta* | KC573961 | - | - | SIS | Cape Verde |
| III | C | 109 FUS | *Caretta caretta* | KC573962 | - | - | SIS | Cape Verde |
| III | C | 110 FUS | *Caretta caretta* | KC573963 | - | - | SIS | Cape Verde |
| III | C | 112 FUS | *Caretta caretta* | KC573964 | - | - | SIS | Cape Verde |
| III | C | 113 FUS | *Caretta caretta* | KC573965 | - | - | SIS | Cape Verde |
| III | C | 114 FUS | *Caretta caretta* | KC573966 | - | - | SIS | Cape Verde |
|  |  |  |  |  | GenBank Accession^b^ |  |  |  |
| Clade | Subclade | Strain^a^ | Host | ITS nrDNA | 28S nrDNA | *RPB2* | Source^c*^ | Origin |
| III | C | 116 FUS | *Caretta caretta* | KC573967 | - | - | SIS | Cape Verde |
| III | C | 117 FUS | *Caretta caretta* | KC573968 | - | - | SIS | Cape Verde |
| III | C | 118 FUS | *Caretta caretta* | KC573969 | - | - | SIS | Cape Verde |
| III | C | 129 FUS | *Caretta caretta* | KC573979 | - | - | SIS | Cape Verde |
| III | C | 130 FUS | *Caretta caretta* | KC573980 | - | - | SIS | Cape Verde |
| III | C | 223 FUS | *Chelonia mydas* | KC574007 | KC573920 | KC574070 | SIS | Ascencion Island |
| III | C | 227 FUS | *Chelonia mydas* | KC574008 | - | - | SIS | Ascencion Island |
| III | C | 228 FUS | *Chelonia mydas* | KC574009 | KC573921 | KC574071 | SIS | Ascencion Island |
| III | C | 230 FUS | *Chelonia mydas* | KC574010 | KC573922 | KC574072 | SIS | Ascencion Island |
| III | C | 233 FUS | *Chelonia mydas* | KC574011 | - | - | SIS | Ascencion Island |
| III | C | 235 FUS | *Chelonia mydas* | KC574013 | - | - | SIS | Ascencion Island |
| III | C | 236 FUS | *Chelonia mydas* | KC574014 | - | - | SIS | Ascencion Island |
| III | C | 237 FUS | *Chelonia mydas* | KC574015 | - | - | SIS | Ascencion Island |
| III | C | 238 FUS | *Chelonia mydas* | KC574016 | - | - | SIS | Ascencion Island |
| III | C | 239 FUS | *Chelonia mydas* | KC574017 | - | - | SIS | Ascencion Island |
| III | C | 240 FUS | *Chelonia mydas* | KC574018 | - | - | SIS | Ascencion Island |
| III | C | 324 FUS | *Dermochelys coriacea* | KC574027 | - | - | AE* | Costa Rica |

^a^Code of the *Fusarium solani* isolates deposited in the fungal collection of the Real Jardín Botánico-CSIC.

^b^ GenBanK accession number of the *F. solani* isolates

^c^ Eggshell without signs of *Fusarium* infection (AE), early infected eggshell (EIS), severely infected eggshell (SIS), infected embryo (IE) and infected plant (IP). The asterisks indicate the eggs that were at early stages of incubation. The other samples correspond to eggs at late stages of incubation.
