# Supplementary material for: Global Distribution of Two Fungal Pathogens Threatening Endangered Sea Turtles
Source: PLoS One. 2014 Jan 21;9(1):e85853. doi: 10.1371/journal.pone.0085853 (PMC3897526; doi:10.1371/journal.pone.0085853)
Supplement: Table S2 — GenBank sequences of the DNA regions: ITS nrDNA, 28S nrDNA, and RPB2 of the isolates of the Fusarium solani species complex included in the phylogenetic analyses. (DOCX) [file pone.0085853.s007.docx]

**Table S2.** GenBank sequences of the DNA regions: ITS nrDNA, 28S nrDNA, and *RPB2* of the isolates of the *Fusarium solani* species complex included in the phylogenetic analyses.

|  |  |  |  |  | GenBank Accession |  |  |  |
| --- | --- | --- | --- | --- | --- | --- | --- | --- |
| Clade | Subclade | Species (anamorph) | NRRL no. | ITS nrDNA | 28S nrDNA | *RPB2* | Source | Origin |
| Outgroup |  | *Fusarium staphyleae* | NRRL22316 | AF178423 | AF178392 | JX171609 | *Staphylea trifolia* | MA, US |
| I |  | *Fusarium illudens* | NRRL22090 | AF178393 | AF178362 | AF178392 | *Beilshmiedia tawa* | New Zealand |
| I |  | *Fusarium plagianthi* | NRRL22632 | AF178417 | AF178386 | JX171614 | *Hoheria glabrata* | New Zealand |
| II |  | *Fusarium phaseoli* | NRRL22276 | EF408520 | EU329668 | JX171608 | *Phaseolus vulgaris* | US |
| II |  | *Fusarium phaseoli* | NRRL31156 | AY220236 | AY220166 | - | *Phaseolus vulgaris* | MI, US |
| II |  | *Fusarium cuneirostrum* | NRRL36024 | AY730905 | AY730875 | *-* | *Phaseolus vulgaris* | Canada |
| II |  | *Fusarium cuneirostrum* | NRRL22158 | AF178396 | L36630 | *-* | *Phaseolus vulgaris* | NY, US |
| II |  | *Fusarium cuneirostrum* | NRRL31104 | AY320195 | - | EU329558 | *Phaseolus vulgaris* | Japan |
| II |  | *Fusarium cuneirostrum* | NRRL22275 | AF178400 | AF178369 | *-* | *Phaseolus vulgaris* | Japan |
| II |  | *Fusarium tucumaniae* | NRRL31096 | EF408523 | GU170656 | EU329557 | *Glycine max* | Argentina |
| II |  | *Fusarium tucumaniae* | NRRL34549 | AY730909 | AY730879 | *-* | *Glycine max* | Argentina |
| II |  | *Fusarium tucumaniae* | NRRL31776 | AY320188 | AY320134 | *-* | *Glycine max* | Brazil |
| II |  | *Fusarium virguliforme* | NRRL22825 | AF178419 | AF178388 | GU170615 | *Glycine max* | IN, US |
| II |  | *Fusarium crassistipitatum* | NRRL36877 | FJ240376 | FJ240376 | FJ240405 | *Glycine max* | Argentina |
| II |  | *Fusarium crassistipitatum* | NRRL31949 | AY320197 | *-* | EU329566 | *Glycine max* | Brazil |
| II |  | *Fusarium brasiliense* | NRRL31757 | AY320184 | *-* | EU329565 | *Glycine max* | Brazil |
| II |  | *Fusarium brasiliense* | NRRL22743 | EF408512 | *-* | EU329525 | *Glycine max* | Brazil |
| II |  | *Fusarium brasiliense* | NRRL22678 | AY320180 | AY320126 | *-* | *Glycine max* | US |
| II |  | *Fusarium sp.* | NRRL22412 | AF178414 | AF178383 | EU329510 | Bark | French Guiana |
| II |  | *Fusarium sp.* | NRRL22387 | AF178403 | AF178372 | EU329505 | Bark | French Guiana |
| II |  | *Fusarium sp.* | NRRL22395 | AF178405 | AF178374 | EU329507 | Bark | Venezuela |
| III |  | *Fusarium lichenicola* | NRRL32434 | DQ094444 | DQ236486 | EF470161 | Human | Germany |
|  |  |  |  |  | GenBank Accession |  |  |  |
| Clade | Subclade | Species (anamorph) | NRRL no. | ITS nrDNA | 28S nrDNA | *RPB2* | Source | Origin |
| III |  | *Fusarium lichenicola* | NRRL28030 | DQ094355 | DQ236397 | EF470146 | Human | Thailand |
| III |  | *Fusarium* f.sp. *cucurbitae* | NRRL22098 | DQ094301 | DQ236243 | EU329489 | Cucurbit | US |
| III |  | *Fusarium* f.sp. *cucurbitae* | NRRL22153 | AF178410 | DQ236344 | EU329492 | Cucurbit | US |
| III |  | *Neocosmospora vasinfecta* | NRRL22166 | DQ094319 | DQ236361 | EU329497 | *Heteroderma glycines* | IL, US |
| III |  | *Neocosmospora vasinfecta* | NRRL22436 | DQ094317 | DQ236259 | EU329511 | Soil | South Africa |
| III |  | *Neocosmospora vasinfecta* | NRRL22468 | DQ094318 | DQ236260 | EU329512 | *Arachis hypogaea* | Guiana |
| III |  | *Fusarium ambrosium* | NRRL20438 | DQ094315 | AF178366 | JX171584 | *Camellia sinensis* | India |
| III |  | *Fusarium ambrosium* | NRRL22346 | EU329669 | EU329669 | EU329503 | *Camellia sinensis* | India |
| III |  | *Fusarium* f.sp. *batatas* | NRRL22400 | DQ094303 | DQ236345 | EU329509 | *Ipomoea batatas* | NC, US |
| III |  | *Fusarium* f.sp. *piperus* | NRRL22570 | AF178422 | AF178391 | EU329513 | *Piper nigrum* | Brazil |
| III |  | *Fusarium* f.sp. *xanthoxyli* | NRRL22163 | AF178394 | AF178363 | EU329496 | *Xanthoxylum piperitum* | Japan |
| III |  | *Fusarium striatum* | NRRL22101 | AF178398 | AF178367 | EU329490 | Cotton duck cloth | Panamá |
| III | A | *Fusarium falciforme* | NRRL31162 | DQ094392 | DQ236434 | EU329560 | Human | TX, US |
| III | A | *Fusarium falciforme* | NRRL32540 | EU329560 | DQ236513 | EU329589 | Human eye | India |
| III | A | *Fusarium falciforme* | NRRL32542 | EU329560 | DQ236515 | EU329590 | Human eye | India |
| III | A | *Fusarium falciforme* | NRRL32754 | DQ094533 | DQ236575 | EU329612 | Turtle adult | FL, US |
| III | A | *Fusarium falciforme* | NRRL28565 | DQ094379 | DQ236421 | EU329555 | Human | TX, US |
| III | A | *Fusarium falciforme* | NRRL32331 | DQ094428 | DQ236470 | EU329577 | Human | TX, US |
| III | B | *Fusarium* f.sp. *pisi* | NRRL45880 | EU329689 | EU329689 | JX171655 | *Pisum sativum* | US |
| III | B | *Fusarium* f.sp. *pisi* | NRRL22278 | DQ094309 | DQ236251 | EU329501 | *Pisum sativum* | US |
| III | B | *Fusarium* f.sp. *cucurbitae* | NRRL43812 | EF453205 | EF453205 | EF470093 | Contact lens solution | NY, US |
| III | B | *Fusarium* f.sp. *cucurbitae* | NRRL22141 | DQ094307 | DQ236249 | EU329491 | *Cucurbita sp* | New Zealand |
| III | B | *Fusarium* f.sp. *cucurbitae* | NRRL32856 | EU329683 | EU329683 | EU329629 | Plaster from ceiling | OH, US |
| III | B | *Fusarium* f.sp*. mori* | NRRL22157 | DQ094306 | DQ094306 | EU329493 | *Morus alba* | Japan |
| III | B | *Fusarium* f.sp*. mori* | NRRL22230 | DQ094305 | DQ236347 | EU329499 | *Morus alba* | Japan |
| III | B | *Fusarium* f.sp. *robiniae* | NRRL22161 | DQ094311 | DQ236353 | EU329494 | *Robinea pseudoacacia* | Japan |
| III | B | *Fusarium* f.sp. *robiniae* | NRRL22586 | DQ094312 | DQ236354 | EU329516 | *Robinea pseudoacacia* | VA, US |
|  |  |  |  |  | GenBank Accession |  |  |  |
| Clade | Subclade | Species (anamorph) | NRRL no. | ITS nrDNA | 28S nrDNA | *RPB2* | Source | Origin |
| III | C | *Fusarium keratoplasticum* | NRRL43649 | EU329687 | EU329687 | EU329639 | Human eye | NV, US |
| III | C | *Fusarium sp.* | NRRL43443 | EF453082 | EF453082 | EF469969 | Human | Italy |
| III | C | *Fusarium keratoplasticum* | NRRL46443 | GU170646 | GU170646 | GU170609 | Human foot | Italy |
| III | C | *Fusarium keratoplasticum* | NRRL46438 | GU170644 | GU170644 | GU170607 | Human toe | Italy |
